# Colonization of β-hemolytic streptococci in patients with erysipelas—a prospective study

**DOI:** 10.1007/s10096-019-03625-9

**Published:** 2019-07-10

**Authors:** Kristina Trell, Sofia Rignér, Marcelina Wierzbicka, Bo Nilson, Magnus Rasmussen

**Affiliations:** 1 Division of Infection Medicine, Lund University, BMC, B14, Tornavägen 10, 22184 Lund, Sweden; 2 Department of Internal Medicine and Emergency Medicine, Skåne University Hospital, Malmö, Sweden; 3 Clinical Microbiology, Lund, Region Skåne Sweden; 4 Department of Laboratory Medicine, Division of Medical Microbiology, Lund University, Lund, Sweden

**Keywords:** Beta-hemolytic streptococci, Erysipelas, *Streptococcus pyogenes*, *Streptococcus dysgalactiae*, Diagnosis

## Abstract

Erysipelas is a common skin infection causing significant morbidity. At present there are no established procedures for bacteriological sampling. Here we investigate the possibility of using cultures for diagnostic purposes by determining the perianal colonization with beta-hemolytic streptococci (BHS) in patients with erysipelas. Patients with erysipelas and a control group of patients with fever without signs of skin infection were prospectively included and cultures for BHS were taken from the tonsils, the perianal area, and wounds. BHS were grouped according to Lancefield antigen, species-determined, and *emm*-typed. Renewed cultures were taken after four weeks from patients with erysipelas and a positive culture for BHS. 25 patients with erysipelas and 25 with fever were included. In the group with erysipelas, 11 patients (44%) were colonized with BHS, ten patients were colonized in the perianal area, and one patient in the throat. In contrast, only one patient in the control group was colonized (*p* = 0.005 for difference). All of the patients with erysipelas colonized with BHS had an erythema located to the lower limb. The BHS were then subjected to MALDI-TOF MS and most commonly found to be *Streptococcus dysgalactiae*. Renewed cultures were taken from nine of the 11 patients with BHS and three of these were still colonized. *Streptococcus dysgalactiae* colonizes the perianal area in a substantial proportion of patients with erysipelas. The possibility of using cultures from this area as a diagnostic method in patients with erysipelas seems promising.

## Introduction

Erysipelas is a prevalent skin infection [1, 2] affecting the upper dermis and clinical signs include an erythema with a sharp demarcation and fever [3, 4]. Erysipelas most commonly affects the leg, followed by the arm and the face [3, 4]. Erysipelas and superficial cellulitis are often used interchangeably but cellulitis typically refers to a skin infection that spreads more diffusely and involves the dermis and subcutaneous tissues [5, 6]. Recurrence is a complication of erysipelas and occurs in 21–29% of the patients, with lymphedema being the most evident risk factor [3, 5, 6].

Erysipelas is a clinical diagnosis and there are no established methods for bacteriological sampling. Blood cultures are positive in about 3–9% of the patients and are only recommended in complicated cases [4, 5, 7]. Cultures from skin lesions when present grow bacteria in up to 70% [4, 5], but whether these bacteria represent the true causative agents is difficult to determine. Bacteriological samplings have also been performed on needle aspirates and skin biopsies from inflamed skin, but rarely identifies pathogenic bacteria [5, 8, 9]. Methods based on PCR have shown similar or even lower yield of positive findings [10, 11]. Serology and direct immunofluorescence have also been used to investigate the causative pathogen [12, 13] but these methods demand additional samplings and are not rapid enough for routine work. Most researchers agree that the major causative pathogens of erysipelas are beta-hemolytic streptococci (BHS) either *Streptococcus pyogenes* (SP, Lancefield group A) or *Streptococcus dysgalactiae* (SD, usually Lancefield group G or C) [5, 14–16].

The colonization of BHS in humans is not fully understood. SP has not been considered to be part of the normal flora in humans, but in a study of patients attending general practice in Denmark, the carriage rate was 2.3% in patients between 15 and 44 years old and 0.6% in patients > 44 years old [17]. Anal and throat carriage of SP in hospital staff have been suggested to be the source of outbreaks of postoperative wound infections [18]. Likewise, transient colonization of the gut and perianal area may develop after throat infections [19]. SD has been recognized as part of the normal flora in the nasopharynx, the genital tract, and the skin. SD is also considered to be colonizer of the intestinal tract [14]. In a study of four cases of erysipelas, perianal carriage of BHS was demonstrated [20].

BHS have previously been considered to be consistently sensitive to penicillin. However, in a recent study, four isolates of SD causing human infection were found to be resistant against penicillin [21]. Increasing resistance rates of BHS against clindamycin and erythromycin have been noted in several studies [22, 23].

The present prospective study was performed to examine if perianal bacteriological sampling could represent a new diagnostic tool. The emergence of antibiotic resistance among BHS emphasizes the need for detection of the causative bacteria.

## Materials and methods

Skåne University Hospital is a tertiary care hospital and the local hospital for approximately 750,000 patients in southern Sweden. Patients aged 18 years or older who presented with erysipelas at the emergency room and the department of Infectious Diseases at Skåne University Hospital between September 2017 and December 2018 were prospectively evaluated for inclusion. The inclusion criteria were sudden onset of an erythema of the skin with sharp borders and presumed bacterial origin in combination with fever, chills, or reduced general condition. Patients were excluded if they (1) had been treated with antibiotics in the last two weeks or if (2) another diagnosis like arthritis, gout, or abscess was more probable. Upon inclusion, a written informed consent was obtained from all participants and they were given detailed printed information. The inclusions were made by an infectious disease physician on duty or by the authors and followed a detailed flowsheet including age, gender, clinical findings, laboratory results, and predisposing factors. Data were obtained by clinical examination and medical history including review of the medical records. Patients seeking medical care with fever caused by other conditions than erysipelas were evaluated for inclusion in the control group and were selected with a case-control ratio 1:1. The inclusion criteria in the control group was solely fever and the two exclusion criteria were (1) the presence of an erythema of the skin with sharp borders or (2) treatment with antibiotics in the last two weeks.

For each culture location, a standard referral was constructed and a special routine was set up for the study at the Department of Clinical Microbiology in Lund, Sweden. The BACTEC FX blood culture system (Becton Dickinson, Franklin Lakes, USA) was used to analyzing blood samples. Patient samples from tonsils, perianal area, and wounds were collected with Liquid Amies Elution Swab (CP480CE ESwab, Copan, Brescia, Italy). The ESwab transport medium and positive blood cultures were cultured on blood agar plates overnight at 37 °C and BHS were identified by the typical appearance of large and solid beta-hemolytic colonies and by grouping into A, C, or G was performed using latex agglutination (Streptex, Remel, Lenexa, KS, USA). Species determination of isolated bacteria was performed with Ultraflextreme matrix-assisted laser desorption/ionization—time of flight mass spectrometry (MALDI-TOF MS) (Bruker Daltonics, Bremen, Germany), using software FlexControl 3.4 and MALDI Biotyper (MBT) Compass 4.1 software, with the MBT Compass Library, DB-7854 MSP as described elsewhere [24]. A score value above 2.0 was considered reliable for the identification to the species level. Isolates of BHS were stored in 10% glycerol and 20% horse serum at − 70 °C and were later subjected to *emm*-typing as described by CDC (https://www.cdc.gov/streplab).

To investigate continuous carriage of BHS, patients with positive cultures from the perianal area and/or the tonsils were scheduled for renewed bacteriological samplings four weeks later. Before the return visit, they were not supposed to have been treated with antibiotic for a period of two weeks.

The study was approved by the Regional Ethics Committee in Lund (no. 2017/514).

Statistical analyses were performed using the Graph Pad Prism version 8. If not otherwise stated, Fisher’s exact test was used to compare categorical variables and Mann-Whitney *U* test used for continuous variables. A *p* value < 0.05 was considered significant.

## Results

### Characteristics of patients

Fifty patients were included, 25 with erysipelas and 25 controls with fever. Patient characteristics are given in Table 1. qSOFA (quick Sequential Organ Failure Assessment) [25] scores were calculated in both groups with similar results. Eleven of the 25 patients with erysipelas had previously had erysipelas compared with two patients in the control group with fever (*p* value = 0.01). None of the patients had experienced any symptoms from the perianal area.

**Table 1:** Characteristics of the patients with erysipelas and with fever

Characteristics	Erysipelas (*n* = 25)	Fever (*n* = 25)	*p* value^a^
Female sex (number of patients)	14	11	0.6
Age, median (IQR)	70 (55–88)	68 (44–80)	0.3
Temperature (° Celsius), median (IQR)^b^	37.5 (37.1–38.6)	38.4 (38.0–39.0)	0.001
Pulse, median (IQR)	89 (80–101)	100 (81–106)	0.22
Respiratory rate, median (IQR)	18 (16–20)	18 (16–20)	0.52
Systolic blood pressure, median (IQR)	130 (120–154)	127 (118–145)	0.59
CRP (mg/L), median (IQR)	50 (13–132)	54 (14–231)	0.75
WBC^c^ count (× 10^9/L), median (IQR)^a^	11.8 (9.3–15.0)	11.3 (8.1–13.7)	0.40
qSOFA^d^ ≥ 2 (number of patients)	1	0	> 0.99
Immunosuppression (> 10 mg Prednisolone/day)	1	1	> 0.99
Previous erysipelas (number of patients)	11	2	0.01
BHS in any location (number of patients)	11	1	0.002
BHS in the perianal area (number of patients)	10	1	0.005

^a^For binary parameters, Fisher’s exact test was used and for continuous variables Mann-Whitney *U* test was used. ^b^*IQR*, interquartile range ^c^white blood cell, ^d^quick Sequential Organ Failure Assessment

### Bacteriological aspects

In the group with erysipelas, 11 patients had positive cultures of BHS (Table 2). Ten patients had BHS in the perianal area of which nine were SD (eight were group G and one was group C, two isolates were lost and could not be species-determined) and one was SP. Three patients had positive throat cultures, four had positive wound cultures, and two had positive blood cultures. One of the patients with fever had a SD in the perianal area. None of the patients in the control group with fever had BHS in the throat. The difference in BHS isolation altogether or in the perianal area was statistically significant (*p* = 0.002 and *p* = 0.005 respectively). Of the 11 patients with a positive culture, we were able to obtain control cultures from nine and three still carried SD in the perianal area. The *emm*-typing performed in nine of the 11 patients confirmed that these patients positive for BHS had the same species and *emm*-type on all locations and occasions. The *emm*-types are given in Table 2.

**Table 2:** The occurrence of BHS among the patients with erysipelas

	First visit				Return visit			
	Tonsil	Perianal	Wound	Blood	Visit	Tonsil	Perianal	Wound
Patient 2	X^1^	GGS^2^ SD stG6.1	^3^	GGS SD stG6.1	Yes	x	x	
Patient 3	GAS SP emm77.0	GAS SP emm77.0	GAS SP emm77.0		Yes	x	x	
Patient 4	x	GGS SD stC74a.0			Yes	x	GGS SD stC74a.0	
Patient 6	GAS SP emm4.0	x	GAS SP emm4.0		Yes	x	x	x
Patient 8	x	GGS SD stG643.0	GGS SD stG643.0		Yes	x	GGS SD stG643.0	x
Patient 10	x	SD GGS stC74.0			Yes	x	x	
Patient 12	GCS SD stG62647.0	GCS SD stG62647.0	x	GCS SD stG62647.0	No			
Patient 14	x	GGS SD stG5420.0	GGS SD stG5420.0	x	No			
Patient 17	x	GGS^4^		x	Yes	x	x	
Patient 18	x	GGS^4^		x	No			
Patient 22	x	GGS^4^			Yes	x	GGS SD stG166b.0	

^1^No growth of BHS. ^2^Abbreviations used are *GGS*, group G *Streptococcus*; *GAS*, group A *Streptococcus* G; *GCS*, group C *Streptococcus*; *SD*, *Streptococcus dysgalactiae*; *SP*, *Streptococcus pyogenes*. ^3^No culture taken. ^4^The sample was not saved for species determination and *emm*-typing

### Characteristics of patients with erysipelas and BHS isolation

Table 3 shows the characteristics of the patients with erysipelas with cultures positive for BHS and the patients where no BHS were isolated. All patients positive for BHS had erysipelas located on the lower limb, compared with the non-positive group where 64% had erysipelas located on the lower limb and 36% located on the upper limb (*p* = 0.05 for difference). In the group with BHS, the systolic blood pressure was found to be significantly lower (*p* = 0.02) and the CRP found to be significantly higher (*p* = 0.002).

**Table 3:** Characteristics of the patients with erysipelas with and without growth of BHS

Characteristics	Erysipelas with BHS (*n* = 11)	Erysipelas without BHS (*n* = 14)	*p* value^a^
Female sex (number of patients)	5	9	0.43
Age, median (IQR)^b^	58 (48–85)	70 (64–80)	0.3
Temperature (° Celsius), median (IQR)^b^	37.4 (36.9–38.9)	37.6 (37.3–38.1)	0.97
Pulse, median (IQR)^b^	91 (82–104)	83 (77–99)	0.46
Respiratory rate, median (IQR)^b^	18 (16–20)	19 (16–21)	0.74
Systolic blood pressure, median (IQR)^b^	121 (105–141)	142 (128–163)	0.02
CRP (mg/L), median (IQR)^b^	138 (44–233)	21 (7–79)	0.002
WBC^c^ count (× 10^9/L), median (IQR)^b^	13.0 (10.1–16.7)	11.4 (8.1–13.1)	0.18
qSOFA^d^ ≥ 2 (number of patients)	1	0	0.44
Area of erythema (cm^2^), median (IQR)^b^	750 (310–1500)	510 (230–1500)	0.35
Erythema located to the lower limb (number of patients)	11	9	0.05
Previous erysipelas (number of patients)	6	5	0.43

^a^For binary parameters, Fisher’s exact test was used and for continuous variables, Mann-Whitney *U* test was used. ^b^Interquartile range, ^c^white blood cell, ^d^quick Sequential Organ Failure Assessment

## Discussion

There is no established bacteriological sampling procedure to help clinicians determine the etiology of erysipelas. Our study shows that patients with erysipelas, especially of the lower limb, are frequently colonized with BHS in the perianal area. These findings are in line with those of a study that was published after the submission of this article [26]. Even if all patients with erysipelas do not have a positive perianal culture, they exceed by far the number of patients with erysipelas and a positive blood culture [4]. Furthermore, patients with BHS in the perianal area tended to have more pronounced signs of inflammation with higher CRP and lower systolic blood pressure than patients with erysipelas without BHS isolation. BHS are known to cause infections in the perianal area, especially in children, but the patients in this study did not experience any symptoms from this region. Perianal cultures of patients with suspected erysipelas could serve at least two purposes. Firstly, a finding of BHS would support the diagnosis of erysipelas and secondly, the isolation of BHS would provide the clinician with guidance to antibiotic therapy. Penicillin should be the first choice for treatment of erysipelas. Recently, four isolates of SD with reduced susceptibility to penicillin have been reported [21]. If resistance becomes a problem in the future, it is very important to determine antibiotic susceptibility of the causative agent to avoid unnecessary broad spectrum antibiotics. Moreover, in patients with allergy to penicillin, alternative antibiotics such as clindamycin or erythromycin might be used and resistance to these antibiotic is relatively common in SD [22, 23].

The majority of BHS found in our study were SD of group G. All patients carrying SD in the perianal area had erysipelas located on the lower limb. These findings are in agreement with recent evidence suggesting that SD is the most common cause of erysipelas and an association between SD and erysipelas of the lower limb [4, 12]. Moreover, SD is prone to cause recurrent erysipelas and cellulitis [4, 12, 27] and in this study, we demonstrate that some patients carry SD in the perianal area also after the conclusion of antibiotic treatment. All these observations would fit with a model where perianal colonization with SD provides a risk for subsequent erysipelas of the lower limb [20]. We speculate that bacteria can dislodge from the perianal area and come into contact with possible breaches of the skin integrity causing erysipelas. If this model is correct, perianal carriage of SD should be considered a risk factor for erysipelas and studies to investigate this will need to be performed. Attempts to reduce or eliminate carriage of SD in the perianal area could also provide a possibility to reduce the risk of recurrence of erysipelas.

A strength of this study is that most of the BHS were both species-determined and *emm*-typed. Since all isolates from a given patient were of the same type, it is likely that a patient is infected and colonized by the same clone though whole genome sequencing would be needed to prove this. Another strength is the prospective study design and the low number of investigators which made the selection of patients very careful. However, enrollment was not continuous as the investigators were more active in including patients during certain periods and several patients had to be excluded since they had already received antibiotics. Moreover, it is feasible that we selected patients with a typical appearance of the infection. The setting of our tertiary care hospital probably also selects for more severely ill patients with comorbidities. It is therefore not evident that our results can be directly applied to other clinical settings. Other limitations to the study are the lack of firm diagnostic criteria for erysipelas, which increases both the risk of including patients without erysipelas and failure to include patients with atypical presentation. The inclusion criterion “reduced general condition” was also somewhat problematic. Moreover, the study is relatively small and was only performed in one center.

In conclusion, this study demonstrates an overrepresentation of perianal colonization of BHS in patients with erysipelas compared with patients with fever caused by other conditions and presents perianal cultures as a new diagnostic tool in cases of erysipelas.
